# The Respiratory Phenotype of Rodent Models of Amyotrophic Lateral Sclerosis and Spinocerebellar Ataxia

**Published:** 2019-11-01

**Authors:** Anna F. Fusco, Angela L. McCall, Justin S. Dhindsa, Logan A. Pucci, Laura M. Strickland, Amanda F. Kahn, Mai K. ElMallah

**Affiliations:** 1 Department of Pediatrics, School of Medicine, Duke University, Durham, NC

**Keywords:** amyotrophic lateral sclerosis, spinocerebellar ataxia, respiration, phrenic, hypoglossal, diaphragm

## Abstract

Amyotrophic lateral sclerosis (ALS) and spinocerebellar ataxia (SCA) are neurodegenerative disorders that result in progressive motor dysfunction and ultimately lead to respiratory failure. Rodent models of neurodegenerative disorders provide a means to study the respiratory motor unit pathology that results in respiratory failure. In addition, they are important for pre-clinical studies of novel therapies that improve breathing, quality of life, and survival. The goal of this review is to compare the respiratory phenotype of two neurodegenerative disorders that have different pathological origins, but similar physiological outcomes. Manuscripts reviewed were identified using specific search terms and exclusion criteria. We excluded manuscripts that investigated novel therapeutics and only included those manuscripts that describe the respiratory pathology. The ALS manuscripts describe pathology in respiratory physiology, the phrenic and hypoglossal motor units, respiratory neural control centers, and accessory respiratory muscles. The SCA rodent model manuscripts characterized pathology in overall respiratory function, phrenic motor units and hypoglossal motor neurons. Overall, a combination of pathology in the respiratory motor units and control centers contribute to devastating respiratory dysfunction.

## Introduction

Amyotrophic lateral sclerosis (ALS) and spinocerebellar ataxia (SCA) are progressive neuromuscular disorders that eventually lead to respiratory insufficiency. Pathology in rodent models of neurodegenerative diseases helps inform mechanisms of respiratory dysfunction [1,2]. These models are important for developing novel therapies that target the entire body including the respiratory system in order to prevent respiratory failure, increase quality of life, and prolong survival in patients [3,4].

ALS is a fatal neurodegenerative disease that causes progressive paralysis. The primary cause of death in ALS patients is respiratory failure [5–10]. Approximately 5–10% of ALS is familial and the remaining patients carry a diagnosis of sporadic ALS. ALS is a heterogeneous disease with a high degree of variability in the pattern of neurodegeneration, age of onset, and rate of progression [6,11,12]. Most patients with ALS only have a life expectancy of 2–5 years, depending on symptom onset [6,13,14]. More than 70% of ALS patients experience limb weakness first and are thus categorized as limb-onset. Patients with limb-onset ALS experience bulbar and respiratory muscle dysfunction later in the disease progression. Bulbar-onset ALS occurs in approximately 25% of patients and is characterized by dysfunction of muscles involved in tongue coordination, speech, swallowing, and vocal cord function [5,6,15]. In contrast to patients with limb-onset ALS, patients with bulbar-onset ALS have a faster disease progression [13,15], since bulbar muscles are essential for airway protection, and proper upper airway patency [16,17]. Therefore, bulbar muscle involvement can lead to recurrent aspiration, infections and upper airway obstruction. Further, in approximately 3–5% of patients, respiratory muscle dysfunction occurs at disease onset [5,18]. Patients with respiratory-onset ALS have a significantly shorter survival time than both bulbar- and limb-onset ALS [18], emphasizing the importance of respiratory muscles in ALS patient survival [19]. Although non-invasive ventilatory support can improve survival and quality of life for many patients, patients with severe bulbar dysfunction do not have a significant survival benefit [14,20,21]. The only other option for patients with severe bulbar dysfunction is invasive ventilatory support which has a substantial negative impact on their quality of life [2,14,22]. Thus, respiratory failure is the leading cause of death in ALS patients, so it is crucial to precisely understand the mechanism by which this disease results in respiratory failure.

Spinocerebellar ataxia (SCA) is a group of rare, progressively neurodegenerative disorders with an autosomal dominant mode of inheritance. Most spinocerebellar ataxias are polyglutamine disorders caused by abnormally long CAG repeats which produce proteins with polyglutamine expansion which are misfolded, toxic, and aggregate in cells. Depending on the protein affected, different cellular pathologies can be present which result in neurodegeneration, especially in the cerebellum [23,24]. Because SCA predominately affects the cerebellum, the disease is defined by ataxia, loss of balance, and dysarthria, however, there are over 40 subtypes that have different symptoms and patterns of disease progression [23,24]. Almost all types of spinocerebellar ataxia have bulbar symptoms, such as difficulty speaking and swallowing. Difficulties swallowing can lead to aspiration and respiratory infection [23]. In addition, a review of patient case studies with a variety of types of SCA revealed that death due to respiratory infection and failure is extremely common [25–31]. Despite the severity of this group of diseases, little research is published on the specific involvement of respiratory dysfunction in SCA patients.

Due to the inevitable and devastating respiratory dysfunction that leads to ventilator dependence and fatality in these neurodegenerative diseases, it is critically important to understand how ALS and SCA affect the respiratory system. This understanding will facilitate the development of novel respiratory-targeted therapies. Transgenic rodents allow researchers to characterize the different components of the respiratory system and identify how each contributes to overall disease pathophysiology.

## Methods and Results of Literature Search

**Figure 1:**
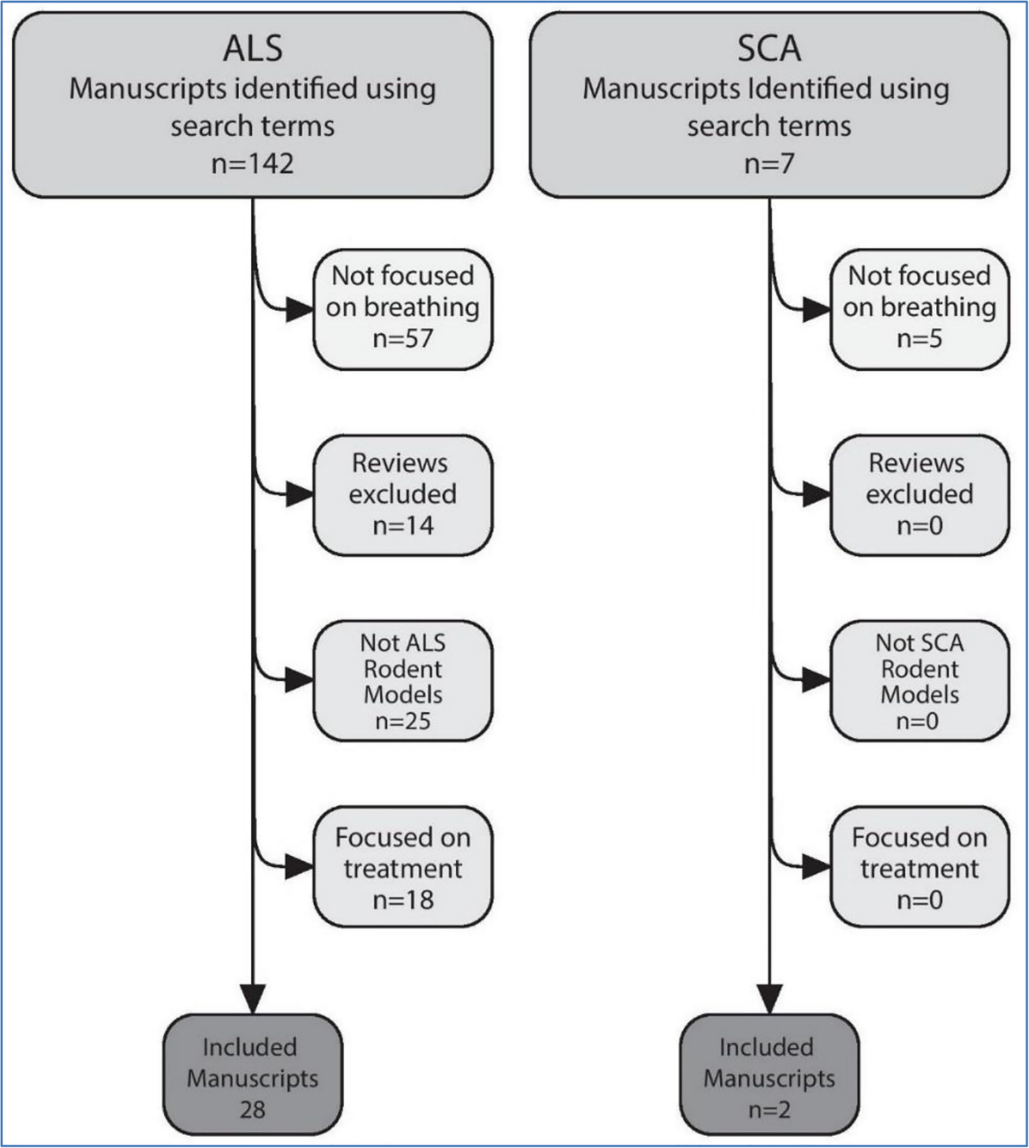
Diagram demonstrating how manuscripts were excluded as a result of the exclusion criteria

See supplement for search terms used and results of the literature search. Figure 1 shows how manuscripts were excluded for each disease, leading to the final set of included manuscripts.

### Amyotrophic Lateral Sclerosis Rodent Models

Mutations in over 40 genes have been associated with ALS. Among those genes, 3–5% of ALS patients harbor mutations in the SOD1 gene [7,12,14]. The SOD1 gene encodes the Cu/Zn superoxide dismutase (SOD1) protein responsible for limiting oxidative damage by reducing toxic reactive oxygen species [11,12,32]. Transgenic rodent models with a mutation that expresses the dominant-negative human SOD1^G93A^ protein are the primary ALS models to date that characterize respiratory dysfunction. Two SOD1^G93A^ mouse models have been used to assess respiratory measures in ALS – the B6SJL-Tg (SOD1*G93A)1Gur/J mouse (hereafter referred to as “Gurney mouse”) [33] and the B6.Cg (SOD1^G93A^)1Gur/J mouse (hereafter referred to as “Wooley mouse”) [34]. The Gurney mouse was developed by the hemizygous insertion of SOD1^G93A^ into Chromosome 12 of a mixed C57BL/6J and SJL mouse. These mice develop paralysis and have a lifespan of approximately 19 weeks. The Wooley mouse was generated by back-breeding the Gurney mouse onto a pure C57BL/6J mouse, to reduce phenotypic variation produced by the mixed B6SJL background.

The Wooley mouse has a longer survival time than the Gurney mouse and survives about 22 weeks [34]. A similar rat model, the NTac:SD-Tg(SOD1^G93A^)L26H rat, has also been developed on a Sprague Dawley background (hereafter referred to as “Howland rat”) [35]. A mouse model which harbors a missense mutation in murine Sod1 that leads to the production of SOD1^G86R^ – the FVB-Tg(Sod1^G86R^)M1Jwg/J mouse (hereafter referred to as “SOD1G86R mouse”)– has also been generated [36]. The Howland rat has an 8-fold greater expression of SOD1^G93A^ relative to endogenous SOD1 expression, which increases throughout the rat’s lifespan. This rat becomes symptomatic at approximately 115 days of age then rapidly declines within two weeks of onset [35]. In addition to mutant SOD1 models, Cho et al. created the SLICK-H::Ranbp2^flox/flox^ mouse (hereafter referred to as “Cho mouse”), a Ranbp2 conditional knockout model, by crossing a Ranbp2 Exon 2 floxed mouse with single-neuron labeling with an inducible Cre-mediated knockout (SLICK) mouse. Cho mice have a very rapid disease progression, immediately presenting with paralysis and respiratory deficits and usually meeting endpoint criteria around 10 days of age [37].

### SOD1 dysfunction

SOD1^G93A^ protein aggregates in neuronal cells and confers a gain of function that is hypothesized to result in the increase of several toxic by-products that lead to oxidative stress. The mutated SOD1 protein forms insoluble protein complexes that aggregate in the cytoplasm of cells in the spinal cord of motor neuron cell cultures, transgenic mice, and humans with SOD1 mutations, and is thought to contribute to neurodegeneration in the SOD1 mutated form of ALS [38]. Howland rats have increased immunostaining for markers of SOD1 in putative phrenic motor neurons as compared to wild type littermates [35]. SOD1 immunostaining has a significant, progressive increase throughout the Howland rats’ lives and is highest at end stage. A gain of function of the SOD1^G93A^ protein and the increase in free radicals is also thought to contribute to an increase in oxidative stress and the progression of ALS [35]. Normally, minimal free radicals such as nitric oxide and superoxides are produced and those that are, are converted to less toxic products by proteins such as SOD1 [39]. In ALS, there is excessive production of nitric oxide, believed to be a result of glutamate excitotoxicity [39]. When nitric oxide levels are elevated and SOD1 activity is normal or reduced, nitric oxide binds to superoxide to form peroxynitrite, which is toxic to the cell.

In addition, the mutated SOD1^G93A^ protein has elevated nitrating activity and catalyzes the addition of peroxynitrite to tyrosine residues, creating nitrotyrosine [39]. Cha et al. studied whether nitrotyrosine is elevated in tissues of the central nervous system in order to determine whether the Gurney mouse has increased NO production which would contribute to increased oxidative stress [39]. Astrocytes throughout the spinal cord of SOD1^G93A^ mice are robustly immunoreactive for nitrotyrosine [39]. These nitrotyrosine immunoreactive astrocytes are localized to the grey matter but are also seen in the brainstem. In the brainstem, nitrotyrosine immunoreactive neurons are observed in important respiratory neurons such as the hypoglossal nucleus, the dorsal motor nucleus of vagus and the nucleus ambiguous [39]. The red nucleus and other structures in the midbrain also contain nitrotyrosine immunoreactive neurons. In contrast, nitrotyrosine immunoreactivity is not observed in the wildtype control mice. These results indicate an increase in nitric oxide production in the transgenic SOD1^G93A^ mice [39]. Nitric oxide is a free radical that has been implicated in ALS pathogenesis and could be a result of glutamatergic excitotoxicity in ALS neurons [40]. The selective increase of nitric oxide in vulnerable neurons in the brain and spinal cord could explain the differential vulnerability seen in ALS patients and the SOD1^G93A^ mouse model as neurons exposed to the increased oxidative stress are more vulnerable to injury and cell death [39,41].

### Overall Respiratory Function

Similar to ALS patients, rodent models of ALS have progressive respiratory dysfunction. A few studies have used whole body plethysmography to assess awake, spontaneous ventilation and forced oscillometry to assess respiratory resistance and compliance [2,7,42]. When awake, spontaneous ventilation is assessed, no significant differences in respiratory parameters are seen between SOD1^G93A^ mice and wild type mice until end stage disease [2,7,42]. However, following the onset of symptoms when the mice are exposed to a respiratory challenge, tidal volume, minute ventilation, and peak inspiratory flow progressively increase compared to wildtype controls [7,42]. Finally, prior to end stage, SOD1^G93A^ mice experience a significant and rapid decline in breathing both at baseline and during a respiratory challenge [2,7,42]. Stoica et al. examined the respiratory mechanics of the SOD1^G93A^ mice and found that there is a significant increase in respiratory resistance and decrease in compliance starting at 13 weeks of age that progresses until there is severe restrictive respiratory disease at end stage [2].

### Phrenic motor neurons and phrenic nerve

Degeneration of phrenic motor neurons is a major contributor to the respiratory decline demonstrated using whole body plethysmography. Phrenic motor neurons innervate the diaphragm–the major muscle of breathing. These neurons are located in the ventral horn of the mid-cervical spinal cord (C3–C5) and their degeneration results in significant respiratory insufficiency. In multiple rodent models of ALS, a decrease in phrenic and C3–C5 motor neuron is documented [42–47]. Phrenic motor neuron loss is progressive and begins before the onset of symptoms [44].

Magnetic resonance imaging (MRI) can noninvasively assess neurodegeneration in the phrenic motor nucleus and the cervical spinal cord [48,49]. MRI demonstrates that the thickness of the cervical spinal cord progressively decreases with disease progression in the SOD1^G86R^ mice. At end stage disease, the cervical spinal cord thickness is also significantly reduced as compared to wildtype. MRI is also used to assess iron content in the spinal cord. Prior autopsy studies have found low levels of iron accumulation in the nervous system [50]. Dysregulation of iron homeostasis is hypothesized to play a role in disease progression and atrophy and is a potential biomarker for diagnosing patients at symptom onset [50]. Iron content in the upper cervical spinal cord is significantly elevated in the SOD1^G86R^ mice at symptom onset but is no longer significantly elevated as compared to wild type at the end stage of the disease due to atrophy of the cervical spinal cord [48]. MRI can also assess spin-spin relaxation time (T2), which is a marker of motor neuron degeneration. The T2 value progressively increases in the upper cervical spinal cord of Gurney mice. In addition, T2 is greater in end stage Gurney mice compared to tg-SOD1 mice overexpressing normal SOD1 protein, and wildtype controls. T2 values of Gurney mice are significantly higher in the central cervical spinal cord and even more significantly elevated in the ventral cervical spinal cord as compared to wildtype mice [49]. Therefore, non-invasive techniques also provide evidence for neurodegeneration in the spinal cord, especially in the regions of the spine that harbour phrenic motor neurons.

Another neurodegeneration marker is glial activation, which is also an indicator of neuroinflammation. PET and CT scans following tail vein injection of the radioligand, 18F-DPA-714, confirm the presence of activated glial cells in the region of the phrenic motor nucleus of the cervical spinal cord of Gurney mice [51]. ^18^F-DPA-714 tags translocator protein (TSPO) expressed in activated glial cells. Uptake of ^18^F-DPA-714 is significantly higher in cervical spinal cords of symptomatic SOD1^G93A^ mice as opposed to wild type SOD1 control mice [51]. Immunohistochemistry staining in the cervical spinal cord for TSPO and Iba1, which is a marker for microglia confirmed the non-invasive PET scan results [51]. TSPO and Iba-1 immunostaining is more robust in SOD1^G93A^ mice as opposed to wild type SOD1 mice, and TSPO colocalizes with Iba-1 when microglia are activated. Together these results demonstrate increased microglial activation in symptomatic SOD1^G93A^ mice, which suggests neuroinflammation and neurodegeneration [51]. In addition, endogenous factors can exacerbate this neuroinflammation and neurodegeneration. For example, hyperstimulation of the phrenic nerve results in a decrease in motor neuron survival and microgliosis [52].

Along with robust spinal and phrenic motor neuron pathologies, phrenic nerve pathology also contributes to atrophy and denervation of the diaphragm that leads to respiratory failure in ALS. At end-stage, Howland rats suffer from a reduction of myelinated fibers in the phrenic nerve. However, there is not a significant difference between the number of myelinated fibers at the proximal and distal segments of the nerve, suggesting that the nerve is not undergoing a “dying back” mechanism. There are rare regenerating clusters of fibers in the proximal segment [47]. SLICK-H::Ranbp2^flox/flox^ mice have a significantly decreased g-ratio, while there is not a significant difference in axon diameter, indicating that the reduction in g-ratio is a result of hypermyelination [37]. Functional deficits accompany these pathological morphologies. For example, Wooley mice have a different diaphragm response when stimulating the diaphragm through the phrenic nerve versus stimulating the diaphragm directly [53]. When stimulating the phrenic nerve, there is a higher time to peak, lower rate of force generation, and lower diaphragm force generated than when stimulating the diaphragm directly or stimulating the phrenic nerve of a wild type mouse. In addition, stimulating through the phrenic nerve increases the susceptibility to intratetanic fatigue [53].

### Diaphragm neuromuscular junction (NMJ) and muscle pathology

The neuromuscular junction (NMJ) between the phrenic nerve and the diaphragm also degenerates during the ALS disease progression. When denervation occurs, the morphology of the NMJ changes. Following denervation of the diaphragm in Gurney mice, the presynaptic terminals disappear and the primary fold flattens [54]. At symptom onset, most presynaptic nerve terminals of SOD1^G93A^ mice have NMJs with similar morphology to wild type. However, a portion of the NMJs have abnormal morphology. Around 40% of presynaptic nerve terminals in SOD1^G93A^ mice have mitochondria that are vacuolated and have pale, empty matrices and disorganized cristae [54]. Gurney mice also have a significant decrease in total synaptic vesicle density at the presynaptic nerve terminal but the size and morphology of those vesicles as well as their distribution in relation to the presynaptic plasma membrane is not significantly different from the wild type control [54]. Ex vivo, these SOD1^G93A^ mice have a significant increase in neurotransmission failure in the diaphragm both at tetanic frequency and firing frequency [53]. Exogenous factors such as hyperstimulation can increase denervation and thus speed up the ALS disease progression. Although Lepore et al. used hyperstimulation of the phrenic nerve as the exogenous factor that increased diaphragm NMJ denervation [52], other exogenous factors such as hypoxia and exposure to toxins have been hypothesized to exacerbate ALS disease progression. These exposures can account for some of the variability in disease onset and the progression of respiratory dysfunction in ALS patients [52].

NMJ pathophysiology changes throughout disease progression and is different during the pre-symptomatic phase than in the symptomatic stage of disease. Rocha et al. conducted intracellular recordings of NMJs by stimulating the phrenic nerve and measuring the evoked activity of the NMJ [55]. During the pre-symptomatic stage, Gurney mice have a significant increase in endplate potential (EPP) amplitude, quantal content, miniature endplate potential (MEPP) amplitude, and giant MEPPs (GMEPP) frequency. There is also a significant decrease in MEPPs rise time as compared to age-matched wildtype. Although the pattern of neuromuscular transmission in the pre-symptomatic SOD1^G93A^ mice significantly differs from age matched wildtype controls, they do not significantly differ from adult wildtype mice, suggesting early maturation of NMJs in Gurney mice. In addition, there is no significant difference between the MEPPs decay time, which indicates that there is not a decrease in acetylcholinesterase [55]. This is confirmed in another study which found that acetylcholinesterase activity in the diaphragm of the Cho mice and control mice are not significantly different [37]. These results provide evidence of disruptions in neurotransmission in ALS rodent models which was confirmed by Rizzuto et al. who found evidence of increased instances of failed neurotransmission in Wooley mice using diaphragm electromyography (EMG) recordings [53,55].

Increase in the pre-symptomatic amplitude of MEPPs indicates a higher permeability of the postsynaptic cell to cations possibly as a result of increased acetylcholine release which is supported by the increase in quantal content of EPPs [55]. Because there is no significant difference in acetylcholinesterase activity [37,55], the dysfunction could be the result of acetylcholine influx into the synaptic cleft [55]. Adenosine A2A receptors fine tune the release of acetylcholine into the synaptic cleft. The role of A2A receptors changes in pre-and post-symptomatic Gurney mouse NMJs. In pre-symptomatic Gurney mice, an agonist for A2A significantly increases the frequency of MEPPs, the amplitude of EPPs, frequency of GMEPPs and quantal content of EPPs, meaning there is a significant increase in the amount of acetylcholine released into the synaptic cleft via an increase in intracellular Ca^2+^ [56]. During the symptomatic stage of disease, a decrease in mean amplitude of EPPs, mean amplitude of MEPPs, and mean rise time of MEPPs in the SOD1^G93A^ NMJs in the symptomatic stage could be a result of disruption in cation influx as a result of decreased sensitivity to acetylcholine [55]. In addition, A2A agonists do not facilitate increases in EPPs amplitude or frequency of MEPPs or GMEPPs providing additional evidence for decreased acetylcholine sensitivity [56].

Pathology in the phrenic nerves and NMJs leads to diaphragm pathology. The diaphragms of Howland rats have dramatic atrophy at end stage as a result of partial denervation of the diaphragm [47]. Only 53.18% of diaphragm muscle fibers are innervated in Gurney mice at end stage [54]. Denervation of the diaphragm leads to decreased mean muscle fiber diameter which could result in the change in MEPP amplitude observed in intracellular recordings of NMJs [54,55]. However, despite significant pathology in phrenic motor neurons, phrenic nerves, neuromuscular junctions, and diaphragm, the diaphragm EMGs of end stage Howland rats do not have a significantly different raw root mean square EMG amplitude during normoxia, maximum chemoreception stimulation (7%CO_2_ and 10.5% O_2_), augmented breath or tracheal occlusion as compared to wild type [47]. This suggests that remaining phrenic motor neurons are compensating by increasing activity. Although the EMG amplitude is normal, the transdiaphragmatic pressure of the Howland rats are significantly lower than in wild type controls during normoxia and tracheal occlusion [47]. The reduced transdiaphragmatic pressure suggests weakness in the diaphragm which could be a result of changes in diaphragm morphology. In addition, respiratory frequency during maximum chemoreception stimulation is significantly lower in Howland rats as compared to wild type. The frequency of augmented breaths is also significantly higher in Howland rats [43]. The compound muscle action potential (CMAP), which is a functional assessment of diaphragm function, is not significantly different in 8 and 12 week old Howland rats as compared to wild type, however, at 16 weeks Howland rats have a decline in CMAP which continues to decline over the next two weeks as rats reach end stage [47]. Hyperstimulation of the phrenic nerves exacerbates the CMAP reduction, suggesting that exogenous factors can worsen diaphragm function [52].

### Hypoglossal motor neurons and nerves

Hypoglossal motor neurons and nerves regulate the shape, stiffness and position of the tongue and thereby maintain airway patency during breathing [17]. ALS patients with involvement of the hypoglossal have bulbar dysfunction and have difficulty swallowing, speaking and maintaining an open airway [15]. Difficulty swallowing can lead to aspiration and increased risk for respiratory infections. In addition, decreased ability to coordinate the tongue during breathing and maintain an open airway can result in airway obstruction during sleep, which can lead to decreased quality of life and respiratory failure [57]. Morphological and physiological changes are present in hypoglossal (XII) motor neurons of rodent models of ALS, although not as consistently as in the phrenic motor neurons. XII motor neurons of Gurney mice have significant vacuolation in the neuropil of axons, dendrites, and glial cells, but not in the motor neuron cell bodies [52,58]. Cytochrome oxidase activity, which can be used as a marker for cellular metabolism, is not significantly different at end stage in Howland rats as compared to wildtype rats [16].

Unlike phrenic motor neurons, XII motor neurons counts do not consistently decrease in number but are still vulnerable to cell death during ALS disease progression. Both Howland rats at 60 days of age [13] and Gurney mice at endpoint [59] have a significant decrease in XII motor neurons. In contrast, Gurney mice at around 117 days of age [47, 51] and Howland rats at 60 days of age [46] have decreased motor neuron counts that are not statistically different from wildtype. Different immunostaining procedures were used by the different researchers which could account for some of this variability, however, the decreased significance in motor neuron loss suggests that the hypoglossal nucleus is not as severely implicated in the rodent SOD1^G93A^ model as in humans [16,47].

Hedlund et al. analyzed gene expression data of Howland rats to determine the similarities between cervical and XII motor neurons, which are vulnerable, and cranial nerve III and IV motor neurons which are not vulnerable [46]. Differential gene expression is found between these “vulnerable” and “not vulnerable” cranial nerves. Peripherin and placental growth factor are predominantly localized to cervical motor neurons which are more vulnerable in ALS [46]. On the other hand, guanine deaminase, IGF-II, Gucy1a3, and early growth response 1 protein are predominantly found in cranial nerve III and IV motor neurons and largely absent in cervical and XII motor neurons [46]. Furthermore, motor neurons pretreated with IGF-II and guanine deaminase prior to glutamate-induced toxicity, have increased motor neuron survival. The presence of IGF-II and guanine deaminase in cranial nerve III and IV could provide protective effects that are absent in the more vulnerable XII and cervical motor neurons, including phrenic motor neurons [46].

As in the cervical spinal cord, MRI images and PET scans show increased neurodegeneration and neuroinflammation in the XII motor nucleus of Gurney mice [49,51]. MRI analysis shows a significant increase in T2 values in the XII nucleus in the medulla that progressively increase, demonstrating progressive neurodegeneration [49]. All time points have significantly elevated T2 values relative to wild type and the T2 values at 90 and 110 days are significantly elevated as compared to tg-SOD1 control. At 90 days of age, the XII motor nucleus also has significantly elevated apparent diffusion coefficient, which is another measure of neurodegeneration [49]. In addition, PET scans and the 18F-DPA-714 tracer to mark activated glial cells reveals increased microglia activation in the XII motor nucleus. Furthermore, there is robust positive staining for TSPO and Iba-1 colocalization demonstrating the presence of activated microglia [51].

When XII neuron viability is assessed following axotomy, there is no significant difference between microglial activation or neuronal viability in 8-week-old Wooley mice and wildtype mice 3 days after XII nerve axotomy, however, the 17-week-old Wooley mice have significantly less activated microglia [60]. However, 21 days after axotomy, neuronal viability is significantly higher and CD3 positive T cells are significantly lower in 17-week-old wildtype mice compared to SOD1^G93A^ mice [60]. In contrast, there is no significant difference between T cell activation in the 8-week-old groups. 17-week-old mice in the wildtype group have increased immunostaining for GDNF, IBF-1, Arginase 1, MC class II and iNOS as compared to 8-week-old wildtype and mSOD1^G93A^ and 17-week-old SOD1^G93A^ mice [60]. Neurotrophic factors such as IGF-1 and GDNF are significantly lower in 17-week-old Wooley mice as compared to 17-week-old wildtype mice. Together, these results suggest that the XII motor nucleus of Wooley mice have a decrease in neuroprotective factors which could lead to increased cell vulnerability [60].

Abnormal development and firing of XII motor neurons could also contribute to disease progression. Johnson et al. studied a mechanism for neurotoxicity using Gurney mice treated with MeHg to hasten the progression of the disease. Significantly higher amounts of Ca^2+^ and another divalent cation like Zn^2+^ are present in SOD1^G93A^ mice as compared to wild type as a result of Ca^2+^ and Zn^2+^ permeable AMPA receptors. Enhanced activation of these AMPA receptors as a result of glutamate-mediated excitotoxicity and enhanced release of glutamate leads to the release of excess Ca^2+^ and Zn^2+^ which are neurotoxic and lead to cell death [61]. Patch-clamp recordings of XII motor neurons in Gurney mice also show hyperexcitability of XII motor neurons. During early stages of life, before the onset of symptoms or cell death, hypoglossal motor neurons of Gurney mice have increased excitability and fire at significantly higher rates than SOD1 wild type mice. There is not a significant difference between groups in action potential firing potential, action potential duration, afterhyperpolarization amplitude, resting membrane potential, input resistance, or mean maximal rate of action potential but Gurney mice have significantly higher action potential amplitudes. By testing the effects of a persistent Na^+^ current inhibitor, Riluzole, van Zundert et al. found that persistent Na^+^ current is a key mechanism to motor neuron hyperexcitability. In addition, Gurney mice have a significantly increased frequency of sAMPA and sIPSC currents and significantly decreased mean sNMDA decay constant. Gurney mice also exhibit early maturation of XII motor neurons [62]. These results demonstrate that disturbances in cation homeostasis and hyperexcitability of motor neurons could lead to increased vulnerability and cell death in the hypoglossal motor nucleus.

### Respiratory control center and accessory muscles

Respiratory neural control centers are essential to the maintenance of proper respiratory rhythm. Gurney mice have a significant decrease in motor neurons in the dorsal motor nucleus of vagus [13,59] which is important in relaying important sensory information from the lungs to respiratory control centers. In addition, Howland rats have a trend towards a decrease in motor neurons in the dorsal motor nucleus of the vagus, but that trend was not significant [13]. The nucleus ambiguus is important in innervating the pharynx and larynx which are also important in maintaining airway patency. Gurney mice have a significant decrease in motor neuron counts in the nucleus ambiguus which is accompanied by astrogliosis, indicative of neuroinflammation and neurodegeneration [59]. Dysfunction in the nucleus ambiguus could contribute to decreased airway patency and bulbar dysfunction [59].

The intercostal motor neurons control the intercostal muscles which are involved in respiratory expiration and are recruited to assist with breathing during an increased respiratory challenge. In ALS, the intercostal muscles are recruited during resting conditions to compensate for decreased diaphragm output. Howland rats have a significant decrease in intercostal motor neuron counts as compared to wild type controls, demonstrating neurodegeneration. The normalized root mean square EMG amplitudes are significantly elevated in the 2^nd^ and 5^th^ intercostal muscles of Howland rats. The increased amplitude of intercostal EMGs indicates that the intercostal muscles of the Howland rats are compensating for the loss of diaphragm function [43].

Other accessory respiratory muscles (ARM) are also recruited to compensate for the degeneration of primary respiratory muscles [42,63]. In mid-symptomatic stages, there are bouts of increased ARM activity which last for one to several breaths [63]. After advanced paralysis, ARM activity is still observed while using the muscles for other tasks, however, ARMs are no longer recruited for breathing. The lack of ARM recruitment results in a rapid decline of ventilation (peak inspiratory flow, tidal volume, minute ventilation, and frequency) [63]. For example, the sternomastoid muscle is an ARM that exhibits significant denervation and atrophy by end stage of Howland rats [16]. Thus, following the loss of ARM innervation or recruitment, there is a rapid respiratory decline leading to respiratory failure [42].

### Spinocerebellar Ataxia

A mouse model of spinocerebellar ataxia 1 (SCA1), the B6.129S-Atxn^tm1Hzo^/J model developed by Dr. Huba Zoghbi, is the only rodent model of any SCA that has been used to study respiration. The Zoghbi mouse has an Atxn1^154Q/2Q^ knock-in mutation inserting 154 CAG repeats in the Ataxin-1 gene [64], as compared to the 38–85 CAG repeats that lead to the clinical form of SCA1 [24]. As in humans, this mutation results in intranuclear aggregation of the mutated Ataxin-1 protein within the brain and spinal cord, causing neurodegeneration. The Zoghbi mouse is typically symptomatic at 20 weeks of age [64]. SCA1 mice have a progressive decrease in tidal volume with a compensatory increase in respiratory rate that preserves minute ventilation. At 6 months of age, the minute ventilation in the SCA1 mice begins to decline which prefaces respiratory failure. There is no significant difference in inter-breath interval irregularity which indicates that regulatory control centers of breathing are still conserved [65]. Jafar-Nejad et al. found similar results in their study of breathing in SCA1 [66]. SCA1 mice do not statistically differ from wildtype littermates at 5 months of age. However, at 33 weeks, SCA1 mice had significantly more shallow breath, indicating a lower tidal volume, and a more rapid respiratory rate [66].

Pathology in the XII nucleus and phrenic motor nucleus are very similar in SCA1 mice [65]. Both undergo motor neuron degeneration and abnormal motor neuron pathology at 6 months. The volume of the soma of both XII and phrenic motor neurons are significantly reduced at 6 months as compared to wildtype mice. In addition, instead of having a normal pyramidal shape the XII and phrenic motor neuron soma are more rounded. Intranuclear aggregates of Atxn1 protein are observed in SCA1 mice beginning at 3 months although the aggregation is not significant until 6 months. Reactive astrocytosis is observed in both the XII and phrenic motor nuclei [65].

SCA1 mice also have significant diaphragm pathology. Denervation, increased esterase activity, and increased small angular contoured muscle fibers are present at 6 months of age. However, there is no necrosis, regeneration of fibers, or fibrosis. These findings indicate that the diaphragm pathologies are not a result of muscle disease. EMG data show that motor unit action potentials have increased amplitudes in SCA1 mice as compared with wildtype which indicates dysfunction in the phrenic motor neurons [65].

## Discussion

**Figure 2:**
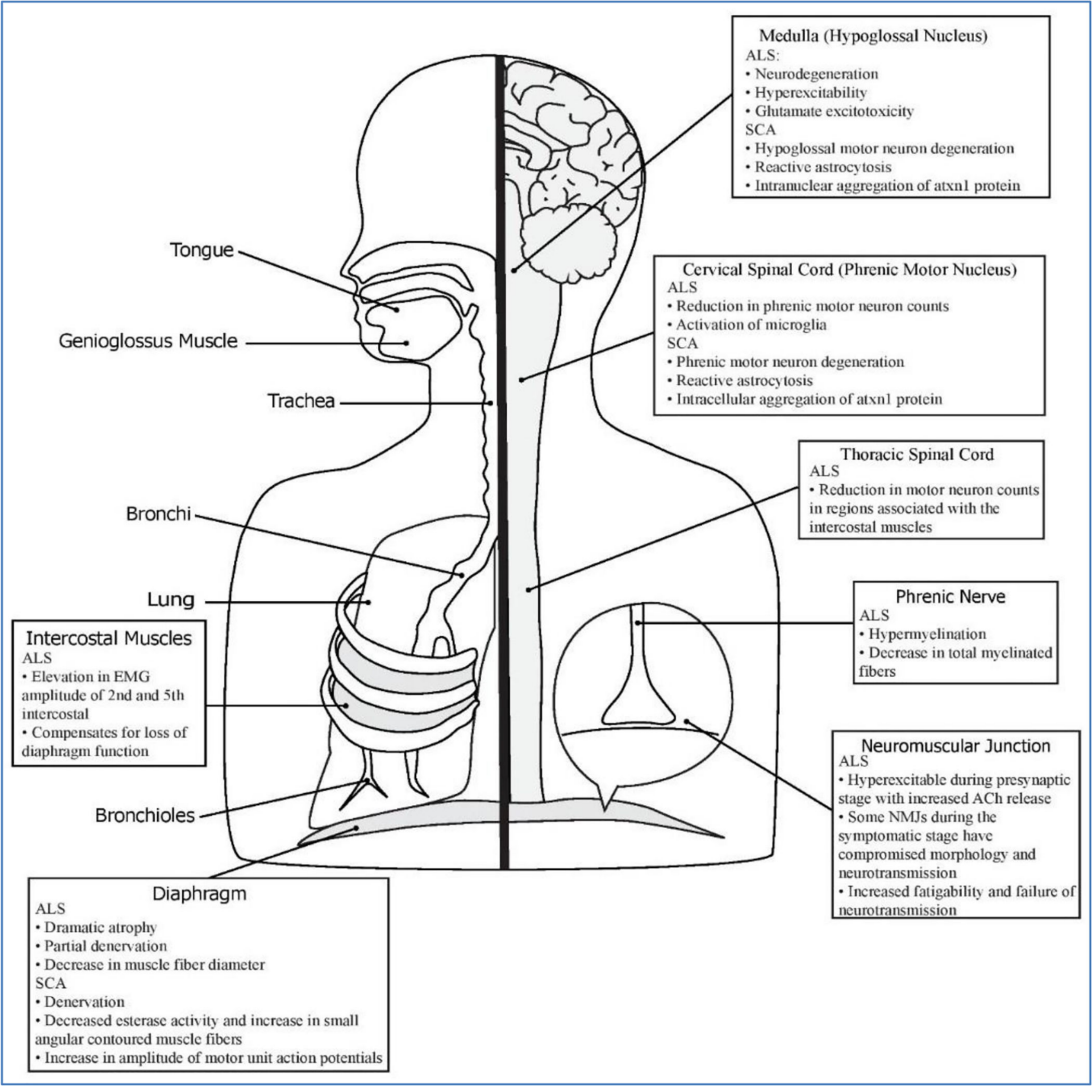
Overview of the respiratory system and the components impacted by ALS and SCA. The ALS manuscripts describe pathology in the phrenic and hypoglossal motor units, respiratory neural control centers, and accessory respiratory muscles. The SCA rodent model manuscripts characterized pathology in phrenic motor units and hypoglossal motor neurons

This review describes the mechanisms of respiratory dysfunction in these rodent models of ALS and SCA (Figure 2 summarizes the respiratory pathology of these rodent models). Whole body plethysmography is a useful tool in assessing parameters of overall respiratory function during awake, spontaneous breathing [67]. Using whole body plethysmography at different time points allows researchers to study the progression of respiratory dysfunction in ALS and SCA. ALS rodent models had a progressive increase in respiratory parameters followed by a rapid decline prior to respiratory failure. The rapid decline of respiratory function is also accompanied by a decline in respiratory compliance and increased respiratory resistance as a complication of restrictive lung disease which occurs in many ALS patients [2]. In contrast, SCA1 mice have a progressive decline in tidal volume and increase in respiratory rate which compensates for minute ventilation until end stage when respiratory function, as in ALS, rapidly declines.

Both ALS and SCA rodent models have neurodegeneration and neuroinflammation in the phrenic motor nucleus and the mid-cervical spinal cord as determined by immunohistochemistry and other noninvasive tools such as magnetic resonance imaging (MRI). The phrenic motor nucleus is located in the mid-cervical spinal cord (C3–C5) and contains the motor neurons that travel via the phrenic nerves to innervate the diaphragm, the major respiratory muscle. ALS rodent models have significantly reduced phrenic motor neuron counts. In addition, noninvasive techniques such as using MRI to analyze iron content, cervical spinal cord volume, and spin-spin relaxation time (T2) support the presence of neurodegeneration in the region of the phrenic motor nucleus in ALS rodent models. Cervical motor neurons in SCA1 rodent models have reduced soma volume and a rounded appearance compared to the typically pyramidal shape. SCA1 rodent models also have increased intranuclear aggregation of Atxn1 protein, suggesting the Atxn1 protein is misfolded and mutated as a result of the CAG repeat mutation. Furthermore, diseases show signs of glial activation in the areas surrounding the phrenic motor pool which is a sign of neuroinflammation and neurodegeneration. In the phrenic motor nucleus, ALS rodents have a progressive increase in microglial activation, whereas rodent models of SCA1 have reactive astrocytosis.

In the ALS rodent models, there is immunohistochemical and electrophysiological evidence of pathologies in the phrenic nerve including hypermyelination and degeneration of myelinated fibers. Intracellular electrophysiological recordings of the diaphragm neuromuscular junctions in ALS rodent models also reveal pathology in neurotransmission and innervation of the postsynaptic diaphragm muscle fiber. Neither the phrenic nerve nor diaphragm neuromuscular junctions were studied in the rodent models of SCA. The diaphragms of ALS and SCA rodents exhibit pathologies consistent with denervation. NMJs of ALS rodent models show evidence of early maturation and increased acetylcholine release in pre-symptomatic NMJs, followed by a portion of the NMJs developing morphological and physiological abnormalities possibly as a result of cation dysfunction. The diaphragm of ALS rodent models has dramatic atrophy and smaller muscle fiber diameters caused by the denervation. SCA1 rodent models have increased esterase activity and an increase in small angular contoured muscle fibers in the diaphragm which also reveal denervation. Diaphragms of SCA1 rodent models do not have necrosis, fibrosis, or fiber regeneration which would be consistent with myopathy but instead have EMG abnormalities consistent with motor neuron dysfunction.

Neural control of airway muscles is also important for breathing. The XII motor neurons help maintain a stable, open airway and coordinate the tongue during breathing. Dysfunction of the XII motor neurons leads to bulbar dysfunction characterized by difficulties swallowing, speaking, and maintaining airway patency, which can lead to respiratory infection or failure [57]. Both ALS and SCA rodent models have degeneration and neuroinflammation in the XII motor nucleus. However, the most common model for ALS, the SOD1^G93A^ model, had inconsistent findings regarding the severity of hypoglossal involvement in the disease progression.

ALS rodent models also have involvement of other respiratory neural control centers such as the dorsal motor nucleus of the vagus nerve which relays sensory information from the lungs to other areas in the brainstem in order to alter breath frequency [68] and the nucleus ambiguus which innervates the pharynx and larynx and helps maintain airway patency [59,69]. In addition, ALS rodent models also have dysfunction in accessory respiratory muscles (ARMs) such as the intercostal muscles. The intercostal motor neurons control the intercostal muscles which normally serve as primary respiratory muscles during expiration and are recruited to assist with breathing during respiratory challenges of exercise [70]. In the beginning of ALS disease progression, ARMs help compensate for respiratory insufficiency but shortly after impairment of their recruitment or neurodegeneration of their neural control centers, respiration rapidly declines, prefacing respiratory failure. Other respiratory neural control centers and ARMs have not yet been studied in SCA rodent models. However, in SCA1, whole-body plethysmography data show a progressive decrease in tidal volume, implying that ARMs are not compensating for respiratory insufficiency as they are in ALS. Both ALS and SCA are devastating neurodegenerative disorders that commonly result in respiratory failure. Understanding how these diseases impact the entire respiratory system is key in being able to research new respiratory targeted therapies to increase the quality and longevity of life in ALS and SCA patients.

## Conclusion

In conclusion, components of the respiratory system such as phrenic motor neurons, the diaphragm, and XII motor neurons are involved in the respiratory pathogenesis of rodent models of ALS and SCA. In addition, the phrenic nerve, neuromuscular junctions, other neural control centers, and accessory respiratory muscles contribute to the progression of respiratory insufficiency in ALS rodent models and may also contribute to pathology in SCA, but this is yet to be studied. Recent discoveries of other causative genes in ALS have resulted in novel mouse models, and novel SCA mouse models are also available. However, the impact of respiratory pathology on these models is unknown. It is necessary to characterize the respiratory phenotype of novel genetic ALS and SCA rodent models in order to increase our understanding of respiratory pathology and disease mechanism. Thus, the respiratory characterization and understanding of breathing pathology is essential in order to test the impact of novel therapeutics on these clinically relevant outcome measures.

## Supplementary Material

Supplement

## References

[1] Nichols NL, Van Dyke J, Nashold L, Satriotomo I, Suzuki M, (2013) Ventilatory control in ALS. Respir Physiol Neurobiol 189: 429–437. 23692930 10.1016/j.resp.2013.05.016 PMC4361018

[2] Stoica L, Keeler AM, Xiong L, Kalfopoulos M, Desrochers K, (2017) Restrictive Lung Disease in the Cu/Zn Superoxide-Dismutase 1 G93A Amyotrophic Lateral Sclerosis Mouse Model. Am J Respir Cell Mol Biol 56: 405–458. 28248134 10.1165/rcmb.2016-0258LE PMC5359540

[3] Stoica L, Todeasa SH, Cabrera GT, Salameh JS, ElMallah MK, (2016) Adeno-associated virus-delivered artificial microRNA extends survival and delays paralysis in an amyotrophic lateral sclerosis mouse model. Ann Neurol 79: 687–700. 26891182 10.1002/ana.24618 PMC5374859

[4] Gibson SJ, Polak JM, Bloom SR, Sabate IM, Mulderry PM, (1984) Calcitonin gene-related peptide immunoreactivity in the spinal cord of man and of eight other species. J Neurosci 4: 3101–3111. 6209366 10.1523/JNEUROSCI.04-12-03101.1984 PMC6564846

[5] Valko K, Ciesla L (2019) A9myotrophic lateral sclerosis. Prog Med Chem 58: 63–117. 30879475 10.1016/bs.pmch.2018.12.001

[6] Al-Chalabi A, Hardiman (2013) The epidemiology of ALS: a conspiracy of genes, environment and time. Nat Rev Neurol 9: 617–628. 24126629 10.1038/nrneurol.2013.203

[7] Tankersley CG, Haenggeli C, Rothstein JD (2007) Respiratory impairment in a mouse model of amyotrophic lateral sclerosis. J Appl Physiol 102: 926–932. 17110520 10.1152/japplphysiol.00193.2006

[8] Kleopa KA, Sherman M, Neal B, Romano GJ, Heiman-Patterson T (1999) Bipap improves survival and rate of pulmonary function decline in patients with ALS. J Neurol Sci 164: 82–88. 10385053 10.1016/s0022-510x(99)00045-3

[9] Aboussouan LS, Khan SU, Meeker DP, Stelmach K, Mitsumoto H (1997) Effect of noninvasive positive-pressure ventilation on survival in amyotrophic lateral sclerosis. Ann Intern Med 127: 450–453. 9313002 10.7326/0003-4819-127-6-199709150-00006

[10] Schiffman PL, Belsh JM (1993) Pulmonary function at diagnosis of amyotrophic lateral sclerosis. Rate of deterioration. Chest 103: 508–513. 8432145 10.1378/chest.103.2.508

[11] Brown RH Jr (1995) Amyotrophic lateral sclerosis: recent insights from genetics and transgenic mice. Cell 80: 687–692. 7889564 10.1016/0092-8674(95)90346-1

[12] Rosen DR, Siddique T, Patterson D, Figlewicz DA, Sapp P, (1993) Mutations in Cu/Zn superoxide dismutase gene are associated with familial amyotrophic lateral sclerosis. Nature 362: 59–62. 8446170 10.1038/362059a0

[13] Kashlan ON, Kashlan BN, Oh SS, McGinley LM, Chen KS, (2015) Histological Bulbar Manifestations in the ALS Rat. Neurodegener Dis 15: 121–126. 25825172 10.1159/000377725

[14] Wijesekera LC, Leigh PN (2009) Amyotrophic lateral sclerosis. Orphanet J Rare Dis 4: 3. 19192301 10.1186/1750-1172-4-3 PMC2656493

[15] Hillel AD, Miller R (1989) Bulbar amyotrophic lateral sclerosis: patterns of progression and clinical management. Head Neck 11: 51–59. 2921111 10.1002/hed.2880110110

[16] Smittkamp SE, Spalding HN, Brown JW, Gupte AA, Chen J, (2010) Measures of bulbar and spinal motor function, muscle innervation, and mitochondrial function in ALS rats. Behav Brain Res 211: 48–57. 20211206 10.1016/j.bbr.2010.03.007 PMC2872192

[17] Fregosi RF, Fuller DD (1997) Respiratory-related control of extrinsic tongue muscle activity. Respir Physiol 110 :295–306. 9407623 10.1016/s0034-5687(97)00095-9

[18] Gautier G, Verschueren A, Monnier A, Attarian S, Salort-Campana E, (2010) ALS with respiratory onset: clinical features and effects of non-invasive ventilation on the prognosis. Amyotroph Lateral Scler 11: 379–382. 20001486 10.3109/17482960903426543

[19] Polkey MI, Lyall RA, Yang K, Johnson E, Leigh PN, (2017) Respiratory Muscle Strength as a Predictive Biomarker for Survival in Amyotrophic Lateral Sclerosis. Am J Respir Crit Care Med 195: 86–95. 27494149 10.1164/rccm.201604-0848OC PMC5214920

[20] Bourke SC, Tomlinson M, Williams TL, Bullock RE, Shaw PJ, (2006) Effects of non-invasive ventilation on survival and quality of life in patients with amyotrophic lateral sclerosis: a randomised controlled trial. Lancet Neurol 5: 140–147. 16426990 10.1016/S1474-4422(05)70326-4

[21] Hobson EV, McDermott CJ (2016) Supportive and symptomatic management of amyotrophic lateral sclerosis. Nat Rev Neurol 12: 526–538. 27514291 10.1038/nrneurol.2016.111

[22] Rowland LP, Shneider NA (2001) Amyotrophic lateral sclerosis. N Engl J Med 344: 1688–1700. 11386269 10.1056/NEJM200105313442207

[23] Sullivan R, Yau WY, O’Connor E, Houlden H (2019) Spinocerebellar ataxia: an update. J Neurol 266: 533–544. 30284037 10.1007/s00415-018-9076-4 PMC6373366

[24] Klockgether T, Mariotti C, Paulson HL (2019) Spinocerebellar ataxia. Nat Rev Dis Primers 5: 24. 30975995 10.1038/s41572-019-0074-3

[25] Ishida C, Komai K, Yonezawa K, Sakajiri K, Nitta E, (2011) An autopsy case of an aged patient with spinocerebellar ataxia type 2. Neuropathol 31: 510–518. 10.1111/j.1440-1789.2010.01176.x 21134000

[26] Adachi T, Kitayama M, Nakano T, Adachi Y, Kato S, (2015) Autopsy case of spinocerebellar ataxia type 31 with severe dementia at the terminal stage. Neuropathol 35: 273–279. 10.1111/neup.12184 25495291

[27] Takao M, Aoyama M, Ishikawa K, Sakiyama Y, Yomono H, (2011) Spinocerebellar ataxia type 2 is associated with Parkinsonism and Lewy body pathology. BMJ Case Rep. 10.1136/bcr.01.2011.3685 PMC3079476 22700602

[28] Gilman S, Sima AA, Junck L, Kluin KJ, Koeppe RA, (1996) Spinocerebellar ataxia type 1 with multiple system degeneration and glial cytoplasmic inclusions. Ann Neurol 39: 241–255. 8967756 10.1002/ana.410390214

[29] Yis U, Dirik E, Kurul SH, Eken AG, Basak AN (2009) Two young sisters with spinocerebellar ataxia type 2 showing different clinical progression of disease. Cerebellum 8: 127–129. 19023636 10.1007/s12311-008-0080-6

[30] Ju H, Kokubu H, Lim J (2014) Beyond the glutamine expansion: influence of posttranslational modifications of ataxin-1 in the pathogenesis of spinocerebellar ataxia type 1. Mol Neurobiol 50: 866–874. 24752589 10.1007/s12035-014-8703-z PMC4821199

[31] Shiojiri T, Tsunemi T, Matsunaga T, Sasaki H, Yabe I, (1999) Vocal cord abductor paralysis in spinocerebellar ataxia type 1. J Neurol Neurosurg Psychiatry 67: 695. 10577039 10.1136/jnnp.67.5.695 PMC1736617

[32] Leclerc N, Ribera F, Zoll J, Warter JM, Poindron P, (2001) Selective changes in mitochondria respiratory properties in oxidative or glycolytic muscle fibers isolated from ^G93A^humanSOD1 transgenic mice. Neuromuscul Disord 11: 722–727. 11595514 10.1016/s0960-8966(01)00240-1

[33] Gurney ME, Pu H, Chiu AY, Dal Canto MC, Polchow CY, (1994) Motor neuron degeneration in mice that express a human Cu,Zn superoxide dismutase mutation. Science 264: 1772–1775. 8209258 10.1126/science.8209258

[34] Wooley CM, Sher RB, Kale A, Frankel WN, Cox GA, (2005) Gait analysis detects early changes in transgenic SOD1(G93A) mice. Muscle Nerve 32: 43–50. 15880561 10.1002/mus.20228 PMC1350398

[35] Howland DS, Liu J, She Y, Goad B, Maragakis NJ, Focal loss of the glutamate transporter EAAT2 in a transgenic rat model of SOD1 mutant-mediated amyotrophic lateral sclerosis (ALS). Proc Natl Acad Sci U S A 99: 1604–1609. 10.1073/pnas.032539299 PMC122237 11818550

[36] Ripps ME, Huntley GW, Hof PR, Morrison JH, Gordon JW. (1995) Transgenic mice expressing an altered murine superoxide dismutase gene provide an animal model of amyotrophic lateral sclerosis. Proc Natl Acad Sci U S A 92: 689–693. 7846037 10.1073/pnas.92.3.689 PMC42685

[37] Cho KI, Yoon D, Qiu S, Danziger Z, Grill WM, (2017) Loss of Ranbp2 in motoneurons causes disruption of nucleocytoplasmic and chemokine signaling, proteostasis of hnRNPH3 and Mmp28, and development of amyotrophic lateral sclerosis-like syndromes. Dis Model Mech 10: 559–579. 28100513 10.1242/dmm.027730 PMC5451164

[38] Turner BJ, Lopes EC, Cheema SS (2003) Neuromuscular accumulation of mutant superoxide dismutase 1 aggregates in a transgenic mouse model of familial amyotrophic lateral sclerosis. Neurosci Lett 350: 132–136. 12972170 10.1016/s0304-3940(03)00893-0

[39] Cha CI, Chung YH, Shin CM, Shin DH, Kim YS, (2000) Immunocytochemical study on the distribution of nitrotyrosine in the brain of the transgenic mice expressing a human Cu/Zn SOD mutation. Brain Res 853: 156–161. 10627320 10.1016/s0006-8993(99)02302-1

[40] Sun W, Funakoshi H, Nakamura T (2002) Overexpression of HGF retards disease progression and prolongs life span in a transgenic mouse model of ALS. J Neurosci 22: 6537–6548. 12151533 10.1523/JNEUROSCI.22-15-06537.2002 PMC6758174

[41] Chung YH, Joo KM, Lee YJ, Cha CI (2003) Immunohistochemical study on the distribution of MnSOD in the central nervous system of the transgenic mice expressing a human Cu/Zn SOD mutation. Brain Res 990: 215–220. 14568347 10.1016/s0006-8993(03)03457-7

[42] Romer SH, Seedle K, Turner SM, Li J, Baccei ML, (2017) Accessory respiratory muscles enhance ventilation in ALS model mice and are activated by excitatory V2a neurons. Exp Neurol 287: 192–204. 27456268 10.1016/j.expneurol.2016.05.033

[43] Seven YB, Nichols NL, Kelly MN, Hobson OR, Satriotomo I, (2018) Compensatory plasticity in diaphragm and intercostal muscle utilization in a rat model of ALS. Exp Neurol 299: 148–156. 29056361 10.1016/j.expneurol.2017.10.015 PMC5951687

[44] Nichols NL, Satriotomo I, Harrigan DJ, Mitchell GS (2015) Acute intermittent hypoxia induced phrenic long-term facilitation despite increased SOD1 expression in a rat model of ALS. Exp Neurol 273: 138–150. 26287750 10.1016/j.expneurol.2015.08.011 PMC4644466

[45] Nichols NL, Vinit S, Bauernschmidt L, Mitchell GS (2015) Respiratory function after selective respiratory motor neuron death from intrapleural CTB-saporin injections. Exp Neurol 267: 18–29. 25476493 10.1016/j.expneurol.2014.11.011 PMC4417059

[46] Hedlund E, Karlsson M, Osborn T, Ludwig W, Isacson O (2010) Global gene expression profiling of somatic motor neuron populations with different vulnerability identify molecules and pathways of degeneration and protection. Brain 133: 2313–2330. 20826431 10.1093/brain/awq167 PMC3139939

[47] Llado J, Haenggeli C, Pardo A, Wong V, Benson L, (2006) Degeneration of respiratory motor neurons in the SOD1 G93A transgenic rat model of ALS. Neurobiol Dis 21: 110–118. 16084734 10.1016/j.nbd.2005.06.019

[48] Grolez G, Kyheng M, Lopes R, Moreau C, Timmerman K, (2018) MRI of the cervical spinal cord predicts respiratory dysfunction in ALS. Sci Rep 8: 1828. 29379040 10.1038/s41598-018-19938-2 PMC5789036

[49] Niessen HG, Angenstein F, Sander K, Kunz WS, Teuchert M, (2006) In vivo quantification of spinal and bulbar motor neuron degeneration in the ^G93A^-SOD1 transgenic mouse model of ALS by T2 relaxation time and apparent diffusion coefficient. Exp Neurol 201: 293–300. 16740261 10.1016/j.expneurol.2006.04.007

[50] Bu XL, Xiang Y, Guo Y (2019) The Role of Iron in Amyotrophic Lateral Sclerosis. Adv Exp Med Biol 1173: 145–152. 31456209 10.1007/978-981-13-9589-5_8

[51] Gargiulo S, Anzilotti S, Coda AR, Gramanzini M, Greco A, (2016) Imaging of brain TSPO expression in a mouse model of amyotrophic lateral sclerosis with (18)F-DPA-714 and micro-PET/CT. Eur J Nucl Med Mol Imaging 43: 1348–1359. 26816193 10.1007/s00259-016-3311-y

[52] Lepore AC, Tolmie C, O’Donnell J, Wright MC, Dejea C, (2010) Peripheral hyperstimulation alters site of disease onset and course in SOD1 rats. Neurobiol Dis 39: 252–264. 20381620 10.1016/j.nbd.2010.03.021 PMC2910141

[53] Rizzuto E, Pisu S, Musaro A, Del Prete Z (2015) Measuring Neuromuscular Junction Functionality in the SOD1(G93A) Animal Model of Amyotrophic Lateral Sclerosis. Ann Biomed Eng 43: 2196–2206. 25631208 10.1007/s10439-015-1259-x PMC4516896

[54] Cappello V, Vezzoli E, Righi M, Fossati M, Mariotti R, (2012) Analysis of neuromuscular junctions and effects of anabolic steroid administration in the SOD1^G93A^ mouse model of ALS. Mol Cell Neurosci 51: 12–21. 22800606 10.1016/j.mcn.2012.07.003

[55] Rocha MC, Pousinha PA, Correia AM, Sebastiao AM, Ribeiro JA (2013) Early changes of neuromuscular transmission in the SOD1(G93A) mice model of ALS start long before motor symptoms onset. PLoS One 8: e73846. 24040091 10.1371/journal.pone.0073846 PMC3764017

[56] Nascimento F, Pousinha PA, Correia AM, Gomes R, Sebastiao AM, (2014) Adenosine A2A receptors activation facilitates neuromuscular transmission in the pre-symptomatic phase of the SOD1(G93A) ALS mice, but not in the symptomatic phase. PLoS One 9: e104081. 25093813 10.1371/journal.pone.0104081 PMC4122437

[57] Lind LA, Murphy ER, Lever TE, Nichols NL (2018) Hypoglossal Motor Neuron Death Via Intralingual CTB-saporin (CTB-SAP) Injections Mimic Aspects of Amyotrophic Lateral Sclerosis (ALS) Related to Dysphagia. Neuroscience 390: 303–316. 30179644 10.1016/j.neuroscience.2018.08.026 PMC6168367

[58] Lever TE, Gorsek A, Cox KT, O’Brien KF, Capra NF, () An animal model of oral dysphagia in amyotrophic lateral sclerosis. Dysphagia 24: 180–195. 19107538 10.1007/s00455-008-9190-z

[59] Ferrucci M, Spalloni A, Bartalucci A, Cantafora E, Fulceri F, (2010) A systematic study of brainstem motor nuclei in a mouse model of ALS, the effects of lithium. Neurobiol Dis 37: 370–383. 19874893 10.1016/j.nbd.2009.10.017

[60] Kawamura MF, Yamasaki R, Kawamura N, Tateishi T, Nagara Y, (2012) Impaired recruitment of neuroprotective microglia and T cells during acute neuronal injury coincides with increased neuronal vulnerability in an amyotrophic lateral sclerosis model. Exp Neurol 234:437–445. 22293437 10.1016/j.expneurol.2012.01.015

[61] Johnson FO, Yuan Y, Hajela RK, Chitrakar A, Parsell DM, (2011) Exposure to an environmental neurotoxicant hastens the onset of amyotrophic lateral sclerosis-like phenotype in human Cu2+/Zn2+ superoxide dismutase 1 ^G93A^ mice: glutamate-mediated excitotoxicity. J Pharmacol Exp Ther 338: 518–527. 21586603 10.1124/jpet.110.174466 PMC3141904

[62] van Zundert B, Peuscher MH, Hynynen M, Chen A, Neve RL, (2008) Neonatal neuronal circuitry shows hyperexcitable disturbance in a mouse model of the adult-onset neurodegenerative disease amyotrophic lateral sclerosis. J Neurosci 28: 10864–10874. 18945894 10.1523/JNEUROSCI.1340-08.2008 PMC3844745

[63] Jensen VN, Romer SH, Turner SM, Crone SA (2017) Repeated Measurement of Respiratory Muscle Activity and Ventilation in Mouse Models of Neuromuscular Disease. J Vis Exp. 10.3791/55599 PMC5565023 28448001

[64] Watase K, Weeber EJ, Xu B, Antalffy B, Yuva-Paylor L, (2002) A long CAG repeat in the mouse Sca1 locus replicates SCA1 features and reveals the impact of protein solubility on selective neurodegeneration. Neuron 34: 905–919. 12086639 10.1016/s0896-6273(02)00733-x

[65] Orengo JP, van der Heijden ME, Hao S, Tang J, Orr HT, (2018) Motor neuron degeneration correlates with respiratory dysfunction in SCA1. Dis Model Mech 11. 10.1242/dmm.032623 PMC5894948 29419414

[66] Jafar-Nejad P, Ward CS, Richman R, Orr HT, Zoghbi HY (2011) Regional rescue of spinocerebellar ataxia type 1 phenotypes by 14-3-3epsilon haploinsufficiency in mice underscores complex pathogenicity in neurodegeneration. Proc Natl Acad Sci U S A 108: 2142–2147. 21245341 10.1073/pnas.1018748108 PMC3033247

[67] Lim R, Zavou MJ, Milton PL, Chan ST, Tan JL, (2014) Measuring respiratory function in mice using unrestrained whole-body plethysmography. J Vis Exp: e51755. 25146417 10.3791/51755 PMC4827935

[68] Yu J (2002) An overview of vagal airway receptors. Sheng Li Xue Bao 54: 451–459. 12506315

[69] Saxon DW, Robertson GN, Hopkins DA (1996) Ultrastructure and synaptology of the nucleus ambiguus in the rat: the semicompact and loose formations. J Comp Neurol 375: 109–127. 8913896 10.1002/(SICI)1096-9861(19961104)375:1<109::AID-CNE7>3.0.CO;2-7

[70] Jaiswal PB, Davenport PW (2016) Intercostal muscle motor behavior during tracheal occlusion conditioning in conscious rats. J Appl Physiol 120: 792–800. 26823339 10.1152/japplphysiol.00436.2015 PMC4824040

