# Supplementary material for: The Respiratory Phenotype of Rodent Models of Amyotrophic Lateral Sclerosis and Spinocerebellar Ataxia
Source: J Neuroinflamm Neurodegener Dis. Author manuscript; Available in PMC 2019 Dec 31. (PMC6938301)
Supplement: Supplement [file NIHMS1062978-supplement-Supplement.docx]

## **Supplement**

## **Methods**

Manuscripts for amyotrophic lateral sclerosis were identified in PubMed using the search term ((((("Amyotrophic Lateral Sclerosis"[MeSH]) OR ((Amyotrophic Lateral Sclerosis)[Title/Abstract] OR (Sclerosis, Amyotrophic Lateral)[Title/Abstract] OR (Motor Neuron Disease, Amyotrophic Lateral Sclerosis)[Title/Abstract] OR (Lou Gehrig's Disease)[Title/Abstract] OR (Lou Gehrig Disease)[Title/Abstract] OR (Lou Gehrigs Disease)[Title/Abstract] OR (Disease, Lou-Gehrigs)[Title/Abstract] OR ((ALS) AND (Amyotrophic Lateral Sclerosis))[Title/Abstract] OR (Gehrig's Disease)[Title/Abstract] OR (Gehrig Disease)[Title/Abstract] OR (Gehrigs Disease)[Title/Abstract]))) AND (("Respiration"[MeSH]) OR (Breath*[Title/Abstract] OR Plethysmography[Title/Abstract] OR Diaphragm[Title/Abstract] OR Phrenic[Title/Abstract] OR Respiratory[Title/Abstract] OR PreBotzinger Complex[Title/Abstract] OR Lung[Title/Abstract] OR Hypoglossal[Title/Abstract]))) AND (("Mice"[MeSH]) OR (Mice[Title/Abstract] OR Mouse[Title/Abstract] OR Rat[Title/abstract] OR rodent[Title/Abstract] OR Murine[Title/Abstract]))) AND English[Language]. Manuscripts for Spinocerebellar Ataxia were identified using the search terms (((((Spinocerebellar Ataxia[Title/Abstract] OR Ataxia, Spinocerebellar[Title/Abstract] OR Ataxias, Spinocerebellar[Title/Abstract] OR Spinocerebellar Ataxia[Title/Abstract] OR SCA (Spinocerebellar Ataxia)[Title/Abstract] OR Spinocerebellar Ataxia Type* [Title/Abstract]))) AND (("Respiration" [MeSH]) OR (Breath*[Title/Abstract] OR Plethysmography[Title/Abstract] OR Diaphragm[Title/Abstract] OR Phrenic[Title/Abstract] OR Respiratory[Title/Abstract] OR PreBötzinger Complex[Title/Abstract] OR Hypoglossal[Title/Abstract]))) AND (("Mice" [MeSH]) OR (Mice[Title/Abstract] OR Mouse[Title/Abstract] OR Rat[Title/abstract] OR rodent[Title/Abstract] OR Murine[Title/Abstract]))) AND English[Language]. The “most recent” function was used to identify manuscripts. Because the goal of the review is to characterize the respiratory phenotype of rodent models of ALS and SCA, manuscripts that did not use rodent models, did not directly study breathing or components of the respiratory system, or focused on assessing possible treatments were excluded. Additionally, review articles were excluded.

## **Results**

**Literature search**

The search terms identified 142 manuscripts for ALS and 7 manuscripts for SCA. After excluding review articles, manuscripts that did not focus on breathing, did not use rodent models, or focused on treatments, 28 manuscripts were included for ALS and 2 manuscripts were included for SCA. Figure 1 shows how manuscripts were excluded for each disease, leading to the final set of included manuscripts.
